# Appendix and Uterus Metastasis of Squamous Cell Carcinoma Arising from Mature Cystic Teratoma of the Ovary

**DOI:** 10.1155/2013/474891

**Published:** 2013-06-13

**Authors:** Gülşah Balık, Işık Üstüner, Recep Bedir, Ülkü Mete Ural, Mehmet Kağıtçı, Emine Seda Güvendağ Güven

**Affiliations:** ^1^Department of Obstetrics and Gynecology, Recep Tayyip Erdoğan University School of Medicine, İslampaşa Mah. Tıp Fakültesi Dekanlığı, Merkez, 53100 Rize, Turkey; ^2^Department of Pathology, Recep Tayyip Erdoğan University School of Medicine, 53100 Rize, Turkey

## Abstract

Mature cystic teratoma of the ovary rarely undergoes malignant transformation. There is no consensus for a treatment modality because of the rarity of the disease. Herein we present a case of squamous cell carcinoma (SCC) arising in a mature cystic teratoma (MCT) in a 66-year-old patient. The patient underwent total hysterectomy and bilateral salpingo-oophorectomy, omentectomy, appendectomy, and bilateral pelvic + paraaortic lymph node dissection. The histopathological examination revealed malignant invasion of the appendix and uterus. The patient, who refused the continuation of treatment initiated with the administration of a single dose of cisplatin, died 5 months later because of the disease. It is imperative that gynecologists consider appendectomy in SCC arising from MCT cases.

## 1. Introduction

Mature cystic teratomas (MCTs) of the ovary, which are a type of germ cell tumors, account for 20%–25 % of all ovarian neoplasms. They are the most common benign germ cell tumors of the ovary in younger women (<45 years). They may be composed of mature or immature tissues deriving from the 3 germ cell layers [1].

Most patients with MCTs are asymptomatic but mass compression effect can lead to pain and abdominal distension. The complications of MCT are rupture, torsion, and malignant transformation [1].

Malignant transformation is very rare in MCT's components (2%). Squamous cell carcinoma (SCC) arising from the ectoderm is the most common type (approximately 80%), which is followed by adenocarcinoma and carcinoids [2, 3].

Herein we present a case of SCC arising in an MCT with appendix metastasis and uterus infiltration in a 66-year-old woman and review the published literature to determine possible risk/prognostic factors and treatments.

## 2. Case Presentation

A 66-year-old multipara patient was referred to our clinic with abdominopelvic mass and abdominal pain. Pelvic examination revealed a large immobile pelvic mass with regular contours extending up to the umbilicus. Ultrasound and computed tomography imaging showed a 20 × 18 cm cystic mass with solid components and calcifications in the left adnexal region extending up to umbilical area with severe ascites. The uterus and right ovarium were normal. The patient's tumor marker profile was as follows: CA-125: 33.1 U/mL (normal <35 U/mL), CA 19-9: 10.54 U/mL (normal <37 U/mL), and β-HcG was negative. The Pap smear being negative for malignancy, and endometrial biopsy revealed atrophy. The patient underwent exploratory laparotomy for ovarian malignancy. 1500 mL serous ascites was noted and used for cytology. The pelvic mass at this time was identified as arising from the left ovary. The posterior surface densely adhered to the pelvic sidewall. The tumor also had massive adhesions to the bladder from its anterior. Total abdominal hysterectomy and bilateral salpingo-oophorectomy were performed. Analysis of a frozen section revealed SCC arising within an MCT. Based on this finding, omentectomy, appendectomy, and bilateral pelvic + paraaortic lymph node dissection were performed. There was no evidence of gross residual tumor.

**Figure 1:**
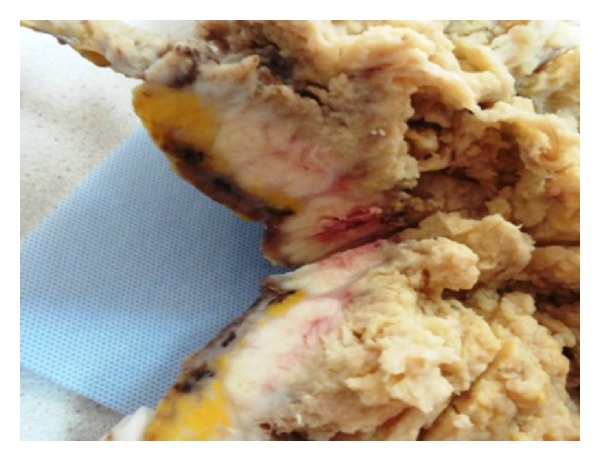
The cyst wall was thickened with a firm mass.

Gross examination of the resulting specimen revealed a solid-cystic mass measured 20 × 15 × 4 cm with a nonintact capsule (Figure 1). The cyst was filled with amorphous debris and the sectioned surface of the cystic mass contained hair, sebaceous material, and focal calcification. The thickness of the cystic wall ranged from 1.5 to 2.4 cm. The tumor invaded the uterine corpus.

**Figure 2:**
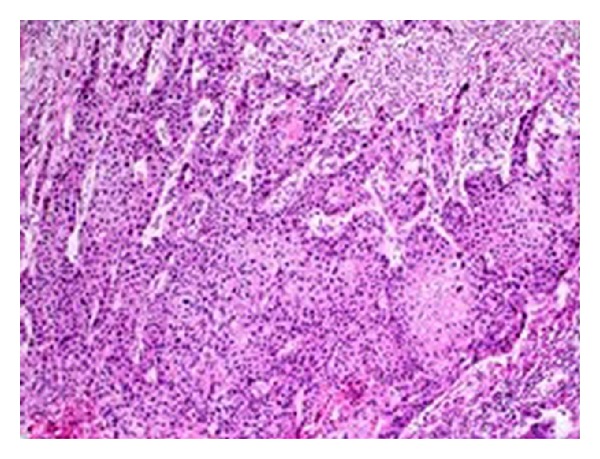
Squamous cell carcinoma, moderately differentiated (H&E ×200).

**Figure 3:**
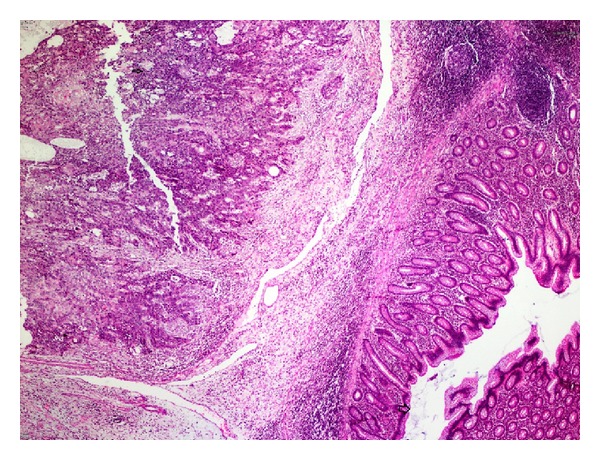
Tumor invasion of appendiceal serosa and muscularis propria (H&E ×40).

The histopathological examination revealed a moderately differentiated SCC arising in an MCT of the ovary. The tumor was composed of solid islands and cords of atypical squamous cells, and in a different side of the cystic wall, teratoma components were seen as a stratified squamous epithelium with adnexal structures (Figure 2). In situ squamous cell carcinoma foci were seen in the tumor. The tumor infiltrated all myometrial layers, the lymphovascular structures, and reached the endometrium. The tumor invaded the appendix vermiformis (Figure 3). The right ovary was normal. There was no tumor in the cervix. Cytological examinations of the ascites and omentum were free of malignant cells. Reactive lymph nodes were detected in the right and left pelvic and paraaortic lymph node dissection materials.

The surgical stage of the case was classified as FIGO stage III. A taxol and carboplatin chemotherapy protocol was planned for the patient. However, the patient refused the planned treatment after the first administration of chemotherapy, and she died five months after the operation.

## 3. Discussion

SCC arising from MCT is a rare pathologic event. Although preoperative diagnosis of MCT is relatively easy, diagnosis of malign transformation is very difficult. Regardless of underlying etiology, SCC of the ovary does not seem to have any pathognomonic features. Malignant transformation is currently diagnosed only by postoperative histopathologic examination in most cases [3]. In our case, frozen section examination facilitated perioperative diagnosis. Thus perioperative frozen section analysis should be done to all relevant cases.

Patient age, tumor size, serum tumor markers, imaging characteristics, and stage constitute risk factors for malignancy arising from MCT [4]. As some studies demonstrated a direct correlation between age (≥45 years) and the occurrence of malignancy, it is prudent to maintain higher awareness of malignancy in MCTs occurring in patients over the age of 45 [5, 6]. Tumor size has also been found to predict malignant transformation. Kikkawa et al. demonstrated that tumors with SCC arising from MCT are sized >9.9 cm and commonly contain areas of hemorrhage and necrosis in most cases [7]. Kim et al. showed that confinement of the disease within the ovary and existence of less than 500 mL ascites may be good prognostic factors [8]. No correlation was found between serum levels of tumor markers (squamous cell antigen (SCC), CA-125, CA19-9, and carcino embryonic antigen (CEA)) and FIGO stage in the SCC from the MCT [1]. However, Mori et al. reported that the combination of the patient's age (>40 years) and serum SCC antigen level (>2.5 ng/mL) was 77% sensitive and 96% specific for malignant transformation [9]. Kikkawa et al. suggested that age and tumor size are better diagnostic factors than serum SCC-antigen and CA 125 level measurements [7]. MCT can be easily diagnosed by imaging studies. However, SCC arising from MCT of the ovary does not have any pathognomonic imaging. Saba et al. showed that the gross appearance of MCTs with malignant transformation was similar to benign MCTs but with a more solid component [10]. Usually this solid component tends to extend transmurally with direct invasion of the pouch of Douglas, uterus, and vagina. Thus, advanced age (66 years), tumor size (20 × 15 × 4 cm), 1500 mL of ascites, and solid component of the cyst were suggestive of malignancy in our case.

Prognostic factors for SCC include capsular invasion/rupture, tumor dissemination, ascites, abdominalpelvic adhesions and tumor types [11]. The gross appearance of nodular, papillary, or ‘‘cauliflower-like” growths invading into the cyst cavities, nodules, or plaques within the cyst walls may also be malignancy indicators [12]. Invasion of the uterus, metastasis into the appendix, ascites, adhesions to bladder, and posterior pelvic sidewall were the bad prognostic factors in our case.

Surgical cytoreduction with proper staging, adjuvant therapy with platinum-based or paclitaxel-based chemotherapy, and concurrent whole pelvic radiation have been recommended as methods of treatment in advanced stage and recurrent disease of SCC arising from MCT [13]. Fertility sparing surgery may be preferred in young patients desiring to retain fertility [13, 14].

If squamous cell lesions of the ovary are detected in a patient, the differential diagnosis should include SCC arising from MCT and metastatic carcinomas. The primary focus of metastasis of squamous cell carcinoma to the ovary is often from uterus and vagina.

Metastasis of the appendix accounts for 4%-5% in early stage cases of epithelial ovarian tumors. Ayhan et al. reported appendix metastasis in 100 of 285 patients with ovarian cancers, and they recommended appendectomy in all ovarian carcinomas for best cytoreduction and surgical staging [14]. Appendectomy is recommended in SCC arising from MCT as the other epithelial ovarian tumors.

The prognosis is poor in SCC arising from MCT. However, early stage of the disease and performing an optimal cytoreductive surgery are good prognostic factors [15].

In conclusion, although malign transformation arising from MCT is extremely rare, clinicians must be aware of this risk before surgery. Because surgical procedure is highly different between benign teratomas and malign transformation arising from MCTs, physicians should consider appendectomy for SCC arising from MCT cases.
